# Supplementary material for: Multiregional single-cell dissection of tumor and immune cells reveals stable lock-and-key features in liver cancer
Source: Nat Commun. 2022 Dec 7;13:7533. doi: 10.1038/s41467-022-35291-5 (PMC9729309; doi:10.1038/s41467-022-35291-5)
Supplement: Supplementary file 1 — Supplementary Information [file 41467_2022_35291_MOESM1_ESM.pdf]

Supplementary Information

Multiregional single-cell dissection of tumor and immune cells reveals stable lock-and-key features in liver cancer

Lichun Ma, Sophia Heinrich, Limin Wang, Friederike L. Keggenhoff, Subreen Khatib, Marshonna Forgues, Michael Kelly, Stephen M. Hewitt, Areeba Saif, Jonathan M. Hernandez, Donna Mabry, Roman Kloeckner, Tim F. Greten, Jittiporn Chaisaingmongkol, Mathuros Ruchirawat, Jens U. Marquardt, Xin Wei Wang

This file contains:
Supplementary Figures 1-17
Supplementary Table 1

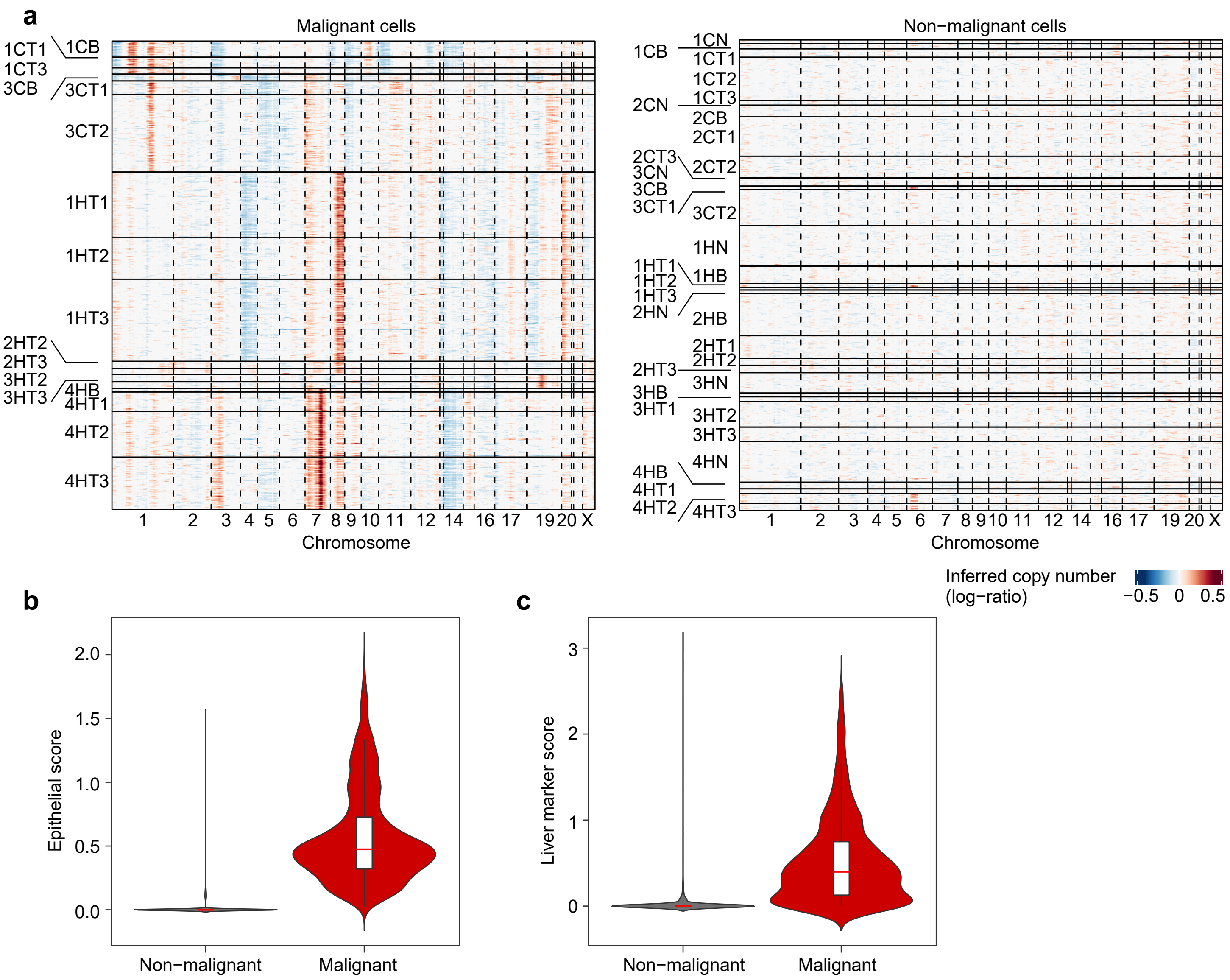

Supplementary Figure 1. Separation of malignant and non-malignant cells.
(a) Inferred large-scale CNVs of malignant cells (left) and non-malignant cells (right). Red, amplifications; blue, deletions. (b and c) Epithelial marker score (b) and liver marker score (c) of malignant cells (n=1,598) and non-malignant cells (n=77,670). Reference cells from normal regions were not included in the violin plot. In the boxplots, the central rectangles span the first quartile to the third quartile, with the segments inside the rectangle corresponding to the median. Whiskers extend 1.5 times the interquartile range.

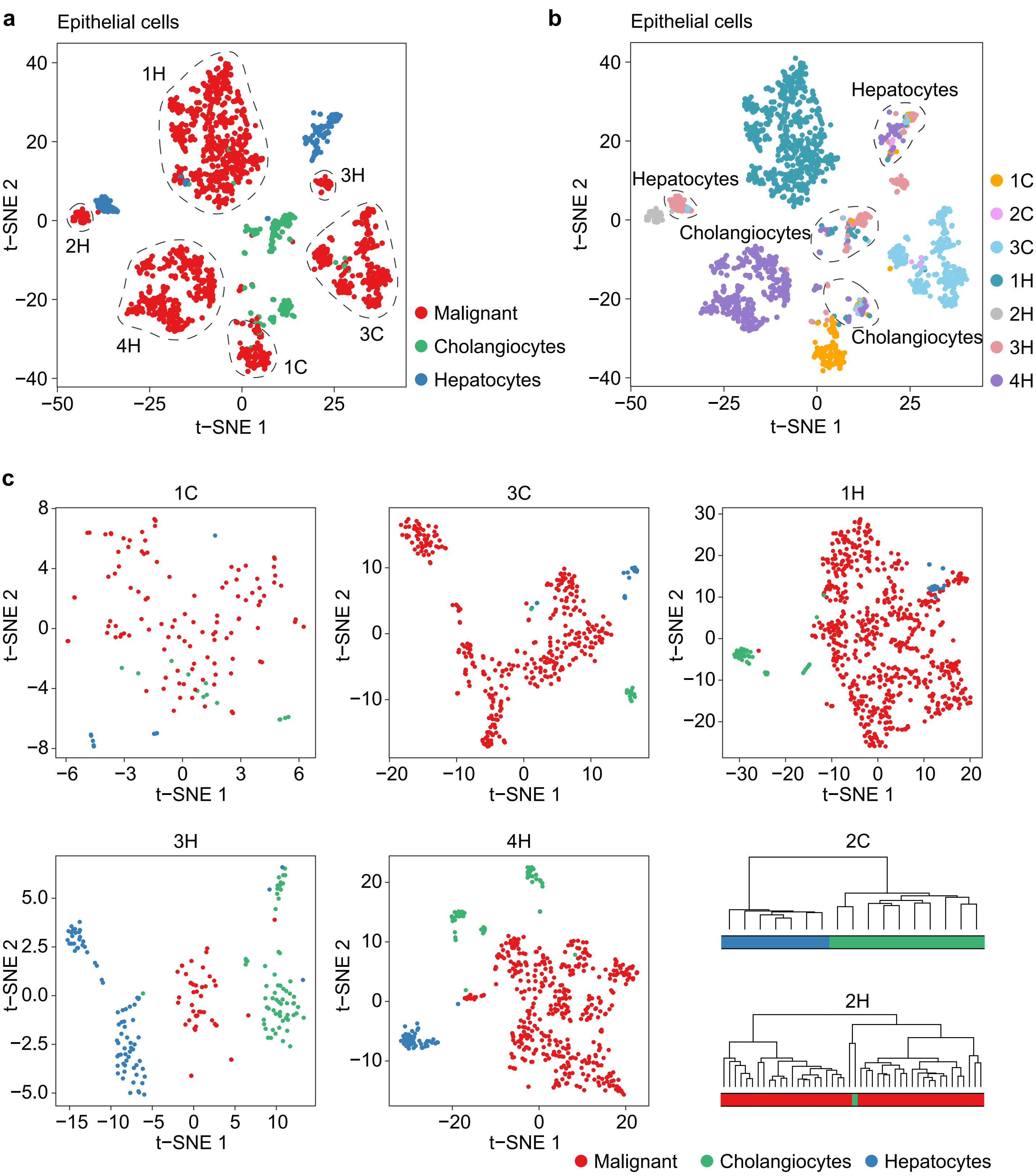

Supplementary Figure 2. Analysis of epithelial cells.
(a and b) t-SNE of epithelial cells from tumor and adjacent normal liver tissues colored by cell types (a) and cases (b). (c) t-SNE or hierarchical clustering of epithelial cells in (a) for each individual case. Due to small number of cells in 2C and 2H, hierarchical clustering was applied due to failure in generating t-SNE plots.

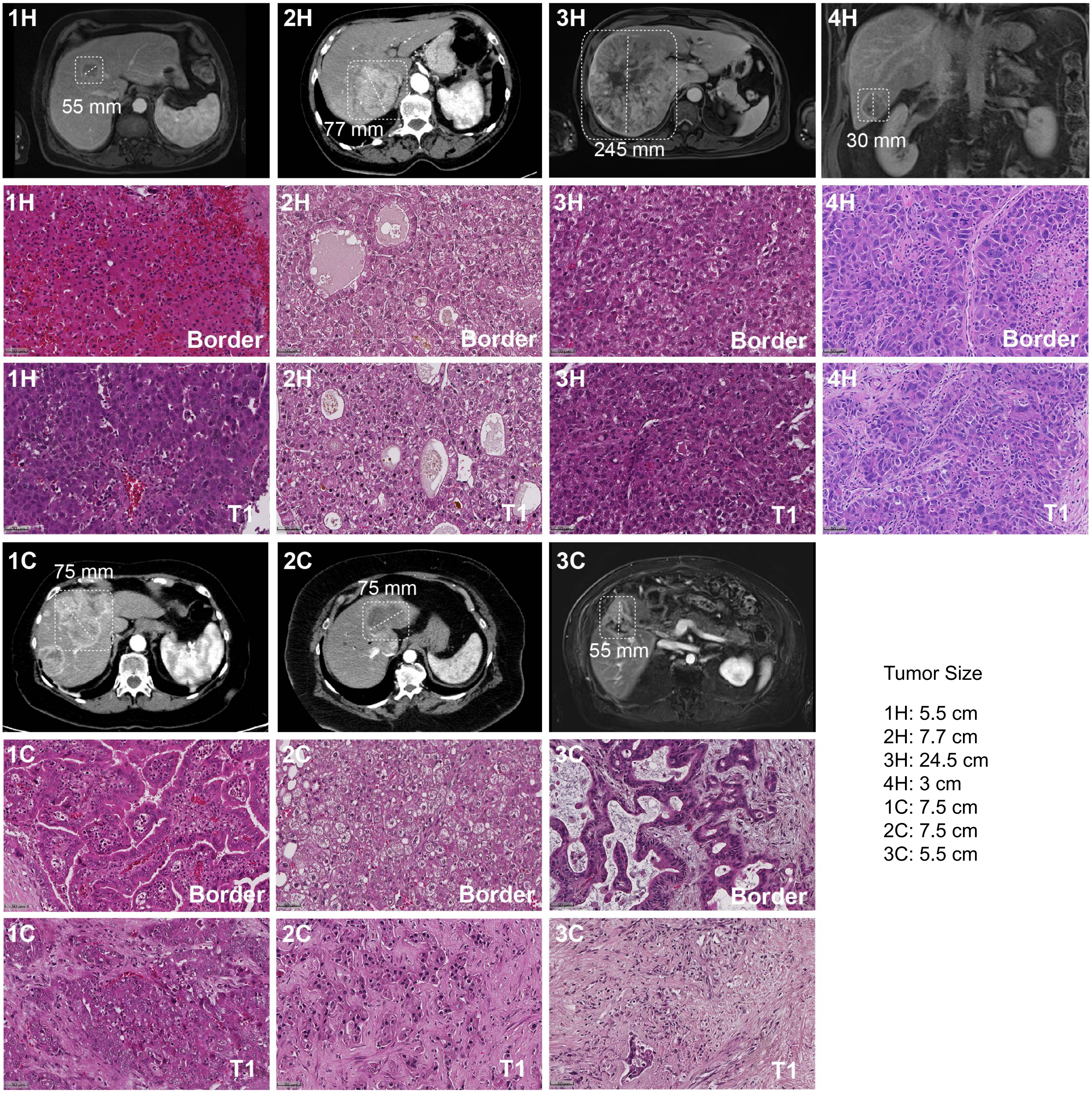

Supplementary Figure 3. Magnetic resonance imaging and histopathology.
Magnetic resonance imaging of all the HCC and iCCA cases and histopathology of tumors collected from tumor border and T1 region. Scale bars, 50 μm.

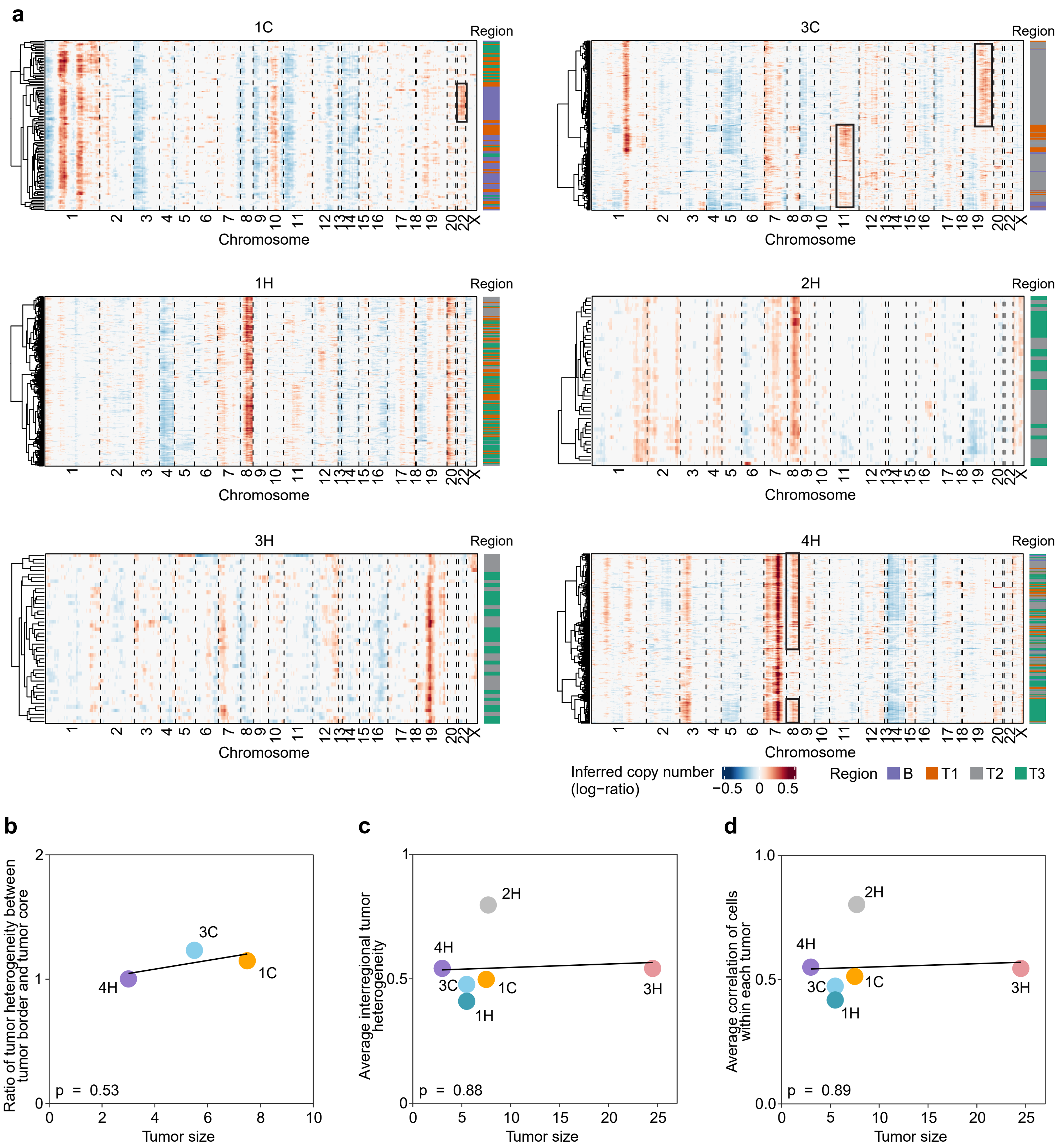

Supplementary Figure 4. Heterogeneity of malignant cells.

(a) Hierarchical clustering of inferred CNVs of malignant cells for each individual case. Examples of clones with distinct CNVs were highlighted using black boxes. (b) The relationship of tumor size and the ratio of tumor heterogeneity (average pair-wise correlation value) between tumor border and tumor core. (c) The relationship of tumor size and the average interregional tumor heterogeneity of each case. (d) The relationship of tumor size and the average pair-wise correlation of all malignant cells within each case. Pearson's correlation test (two-sided) was used in (b-d) to indicate the statistical significance of the relationship. See also Fig. 1f.

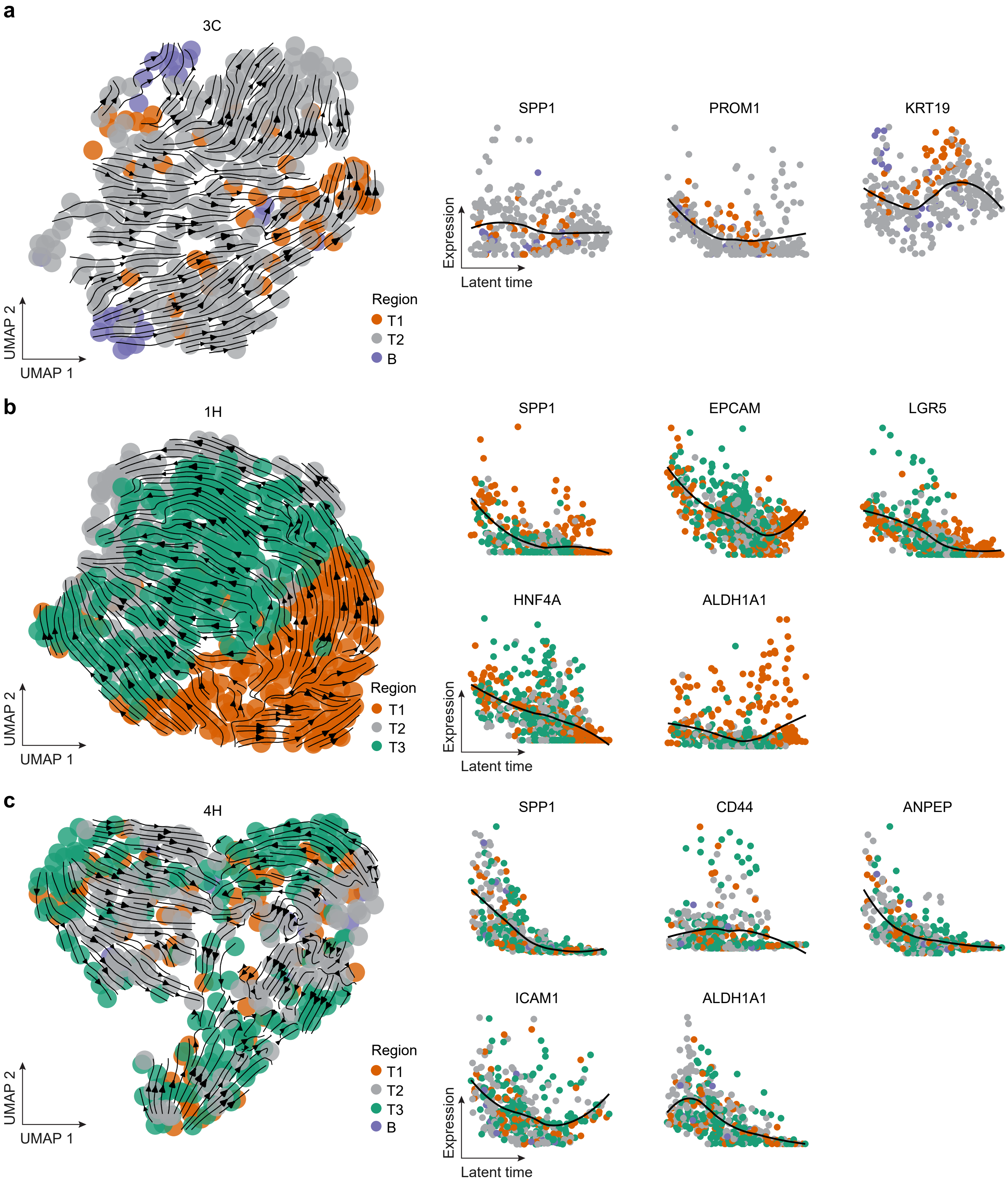

Supplementary Figure 5. Multiregional tumor cell trajectory.
(a-c) RNA velocity of malignant cells from multiple tumor regions and expression of several marker genes (cell stemness-related marker genes and SPP1) along cellular latent time of case 3C (a), 1H (b) and 4H (c).

a

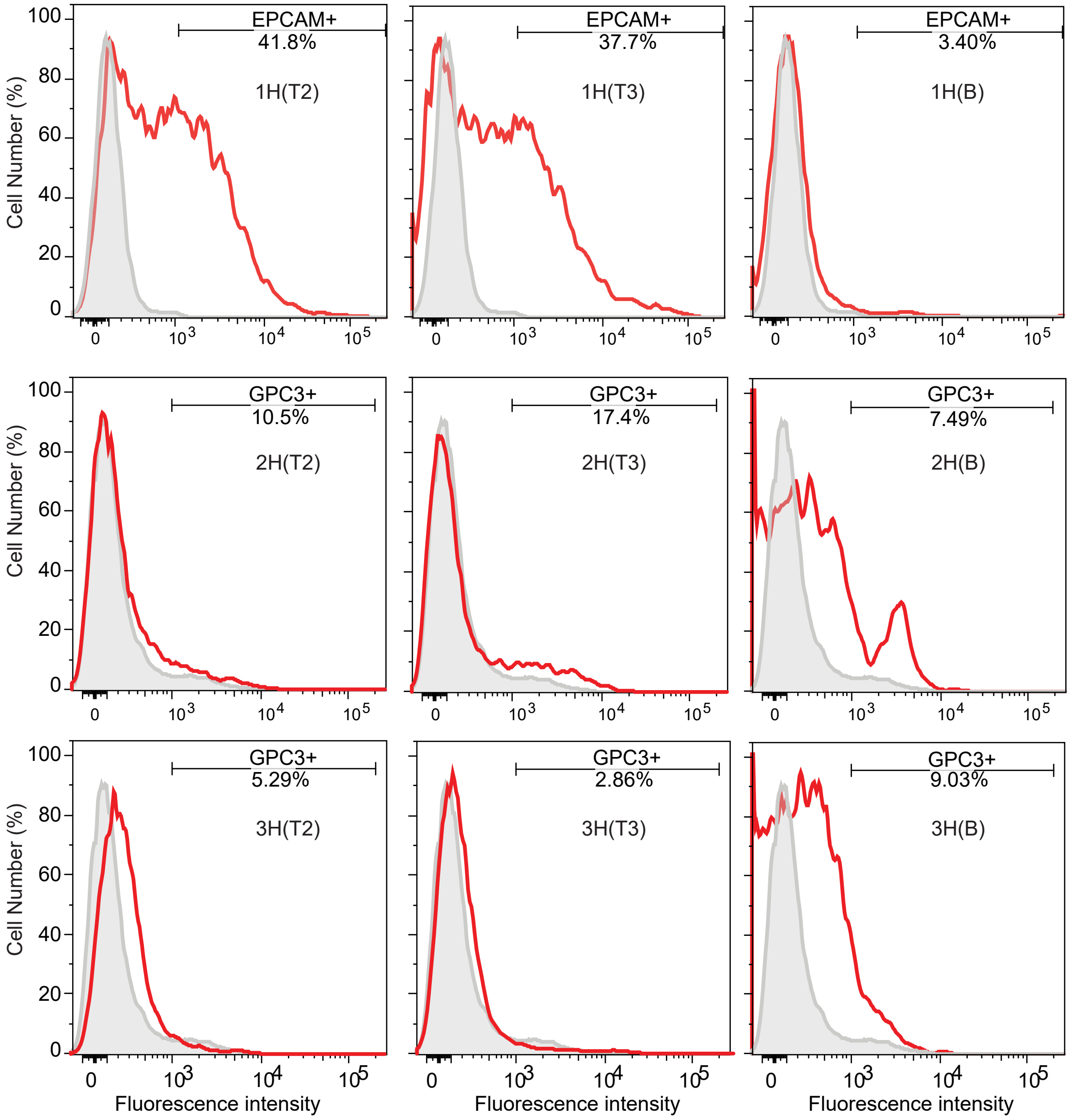

b

Case	1H			2H			3H		
Sample	1H(T2)	1H(T3)	1H(B)	2H(T2)	2H(T3)	2H(B)	3H(T2)	3H(T3)	3H(B)
Estimated total number of cells	5,856	15,575	9,068	9,052	4,423	3,190	3,358	9,158	9,134

c

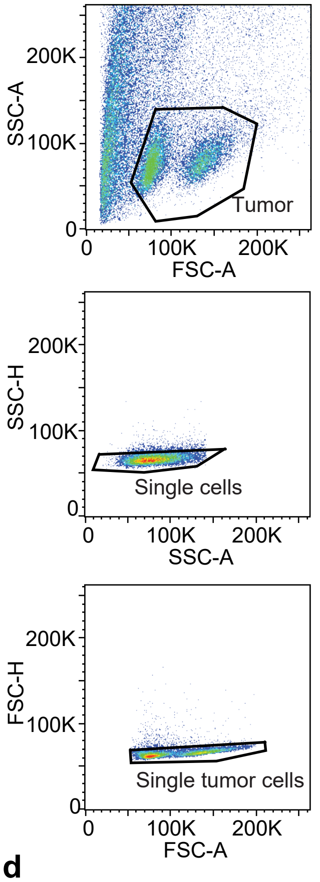

d

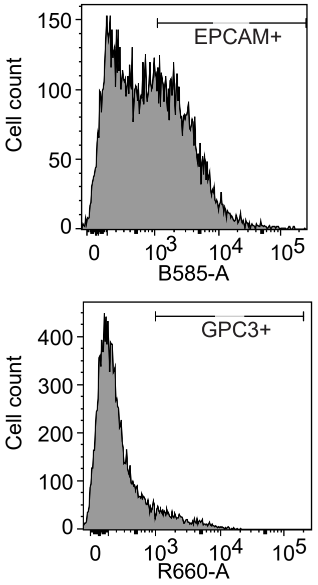

Supplementary Figure 6. FACS of EPCAM+ or GPC3+ cells.

(a) Distribution of EPCAM+ cells from different tumor regions of 1H (first row) and GPC3+ cells from multiple regions of 2H and 3H (second and third rows). The percentage of EPCAM+ or GPC3+ cells was indicated on figures. (b) Estimated total number of cells from the samples in (a). (c and d) Cell gating strategy: cells were sequentially gated (c, from top to bottom) with indicated scatters to obtain a uniform population of single tumor cells by excluding debris, dead cells and clusters, and then gated with cells stained using antibody isotype control to get EPCAM and GPC3 signal as shown in representative samples (d).

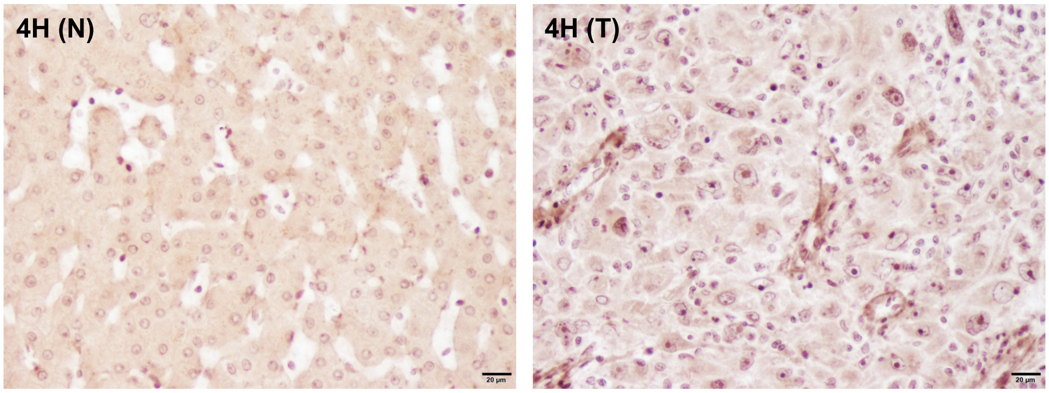

Supplementary Figure 7. Immunohistochemistry of SPP1.
Immunohistochemistry of SPP1 in non-tumor (left) and tumor (right) tissues from case 4H. Scale bars, 20 μm.

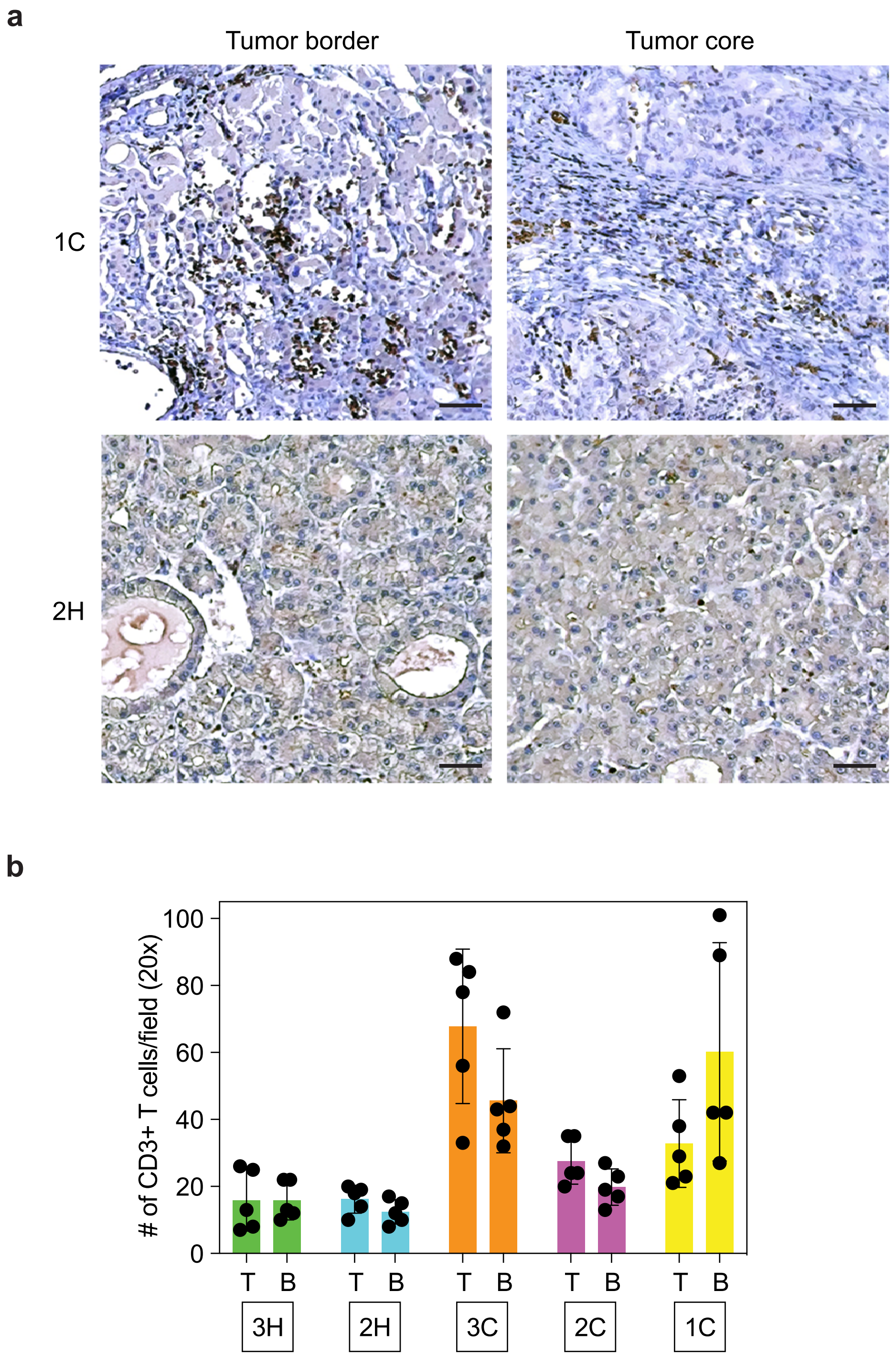

Supplementary Figure 8. Immunohistochemistry of CD3+ T cells.
(a) Representative images of CD3 staining of tumor border and tumor core regions for cases of 1C and 2H. Scale bars, 50 µm. (b) Number of CD3+ positive cells per field of view (~0.2 mm). A total of 5 fields of view were randomly selected for each tumor region. Error bar, mean ± standard deviation.

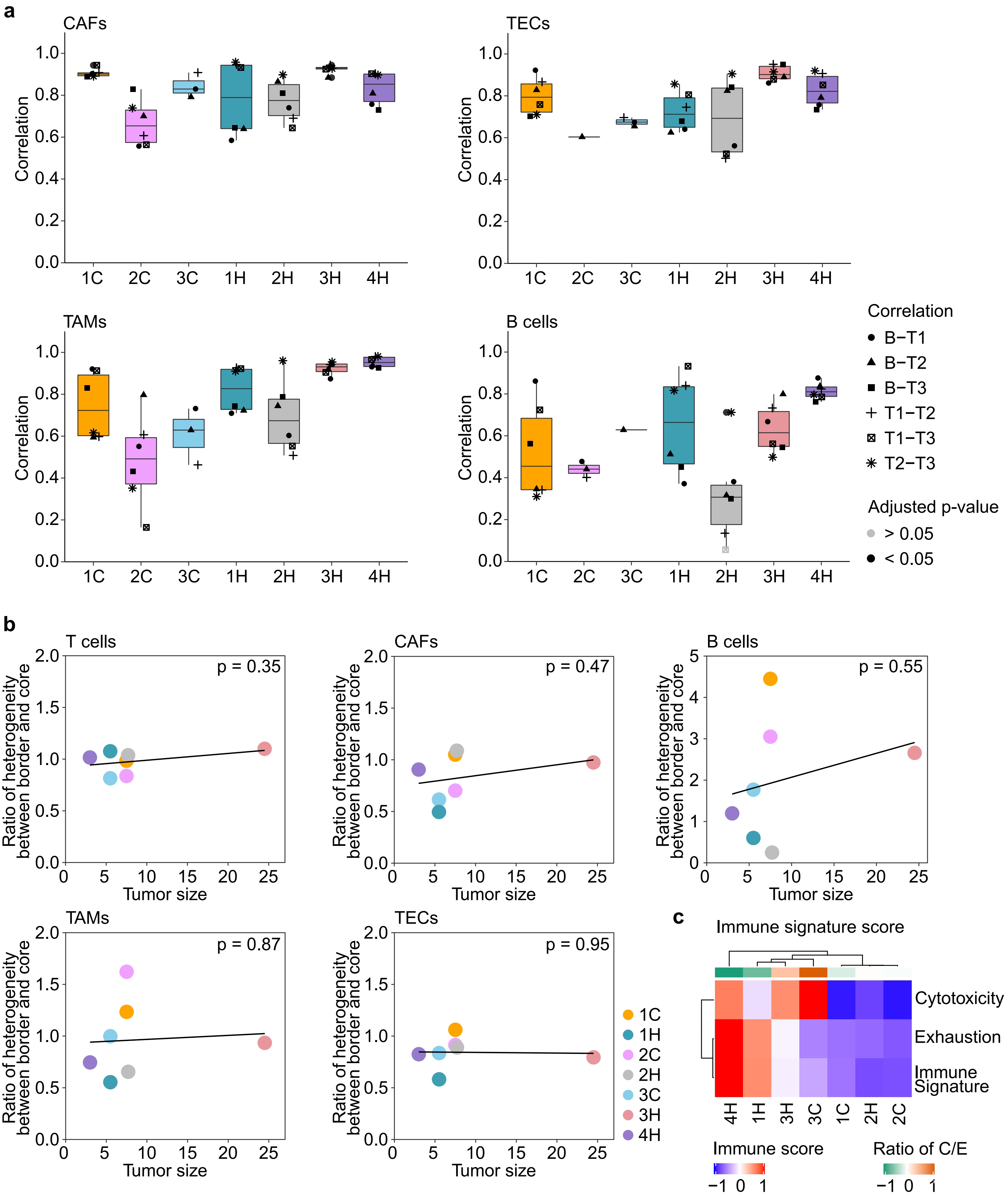

a
CAFs
TECs
TAMs
B cells
Correlation
1C
2C
3C
1H
2H
3H
4H
Correlation
B−T1
B−T2
B−T3
T1−T2
T1−T3
T2−T3
Adjusted p-value
> 0.05
< 0.05
b
T cells
CAFs
B cells
TAMs
TECs
p = 0.35
p = 0.47
p = 0.55
p = 0.87
p = 0.95
Ratio of heterogeneity between border and core
Tumor size
c
Immune signature score
Cytotoxicity
Exhaustion
Immune Signature
Immune score
Ratio of C/E

Supplementary Figure 9. Analyses of non-malignant cells.

(a) Correlation of each non-malignant cell type (i.e., CAFs [n=1,626], TECs [n=1,176], TAMs [n=6,602] and B cells [n=1,774]) between different tumor regions of each individual case. Pearson's correlation test (two-sided) was applied. In the boxplots, the central rectangles span the first quartile to the third quartile, with the segments inside the rectangle corresponding to the median. Whiskers extend 1.5 times the interquartile range. (b) The relationship of tumor size and the ratio of non-malignant cell heterogeneity (average correlation value) between tumor border and tumor core. Pearson's correlation test (two-sided) was used to indicate the statistical significance of the relationship. (c) Immune score of each case. Cytotoxic, exhaustion and overall immune scores were calculated. C/E, the ratio of cytotoxic and exhaustion scores.

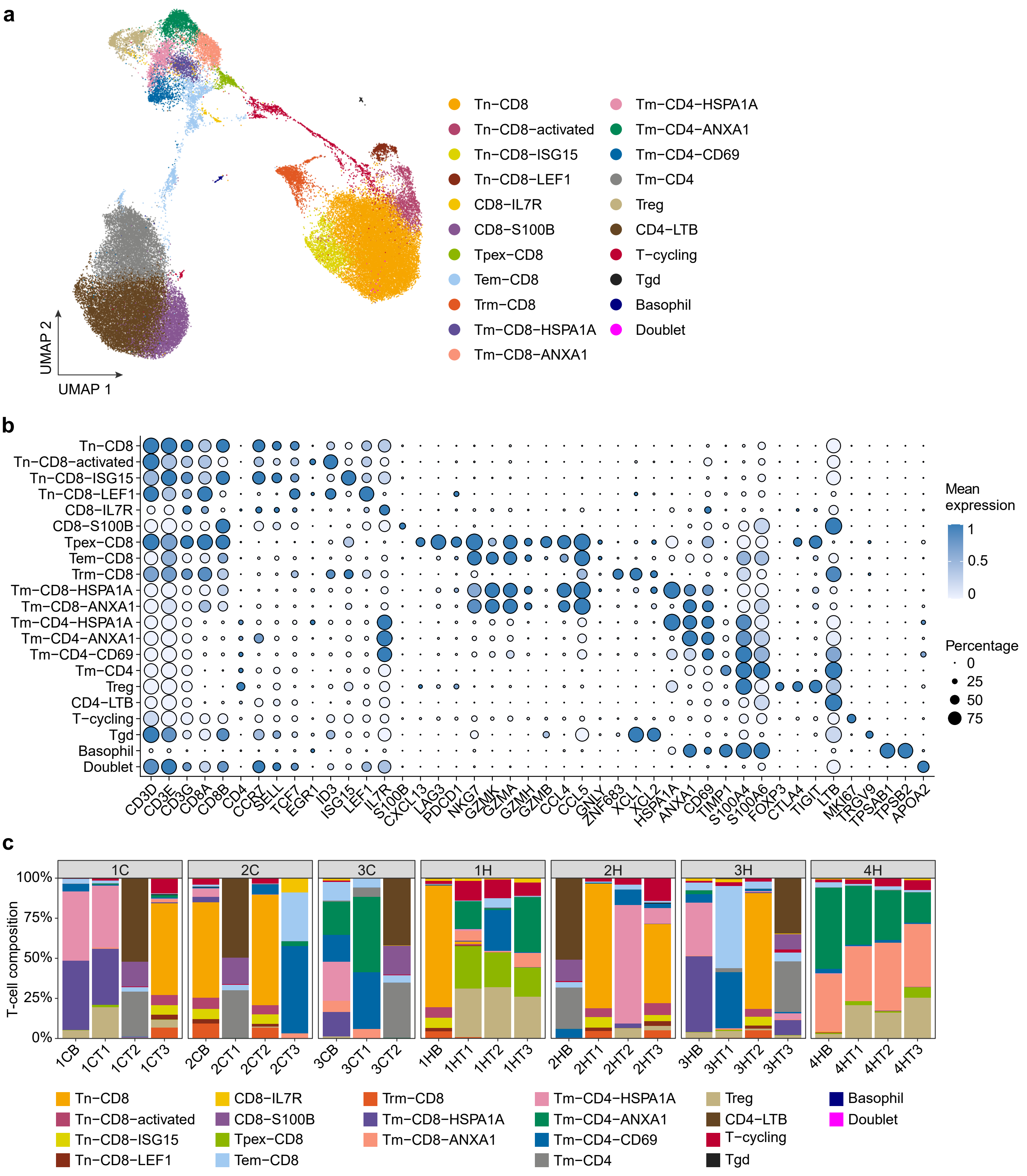
a
UMAP 1
UMAP 2
Tn−CD8
Tn−CD8−activated
Tn−CD8−ISG15
Tn−CD8−LEF1
CD8−IL7R
CD8−S100B
Tpex−CD8
Tem−CD8
Trm−CD8
Tm−CD8−HSPA1A
Tm−CD8−ANXA1
Tm−CD4−HSPA1A
Tm−CD4−ANXA1
Tm−CD4−CD69
Tm−CD4
Treg
CD4−LTB
T−cycling
Tgd
Basophil
Doublet
b
Tn−CD8
Tn−CD8−activated
Tn−CD8−ISG15
Tn−CD8−LEF1
CD8−IL7R
CD8−S100B
Tpex−CD8
Tem−CD8
Trm−CD8
Tm−CD8−HSPA1A
Tm−CD8−ANXA1
Tm−CD4−HSPA1A
Tm−CD4−ANXA1
Tm−CD4−CD69
Tm−CD4
Treg
CD4−LTB
T−cycling
Tgd
Basophil
Doublet
CD3D CD3E CD3G CD8A CD8B CD4 CCR7 SELL TCF7 EGR1 ID3 ISG15 LEF1 IL7R S100B CXCL13 LAG3 PDCD1 NKG7 GZMK GZMA GZMH GZMB CCL4 CCL5 GNLY ZNF683 XCL1 XCL2 HSPA1A ANXA1 CD69 TIMP1 S100A4 S100A6 FOXP3 CTLA4 TIGIT LTB MKI67 TRGV9 TPSAB1 TPSB2 APOA2
Mean expression
1
0.5
0
Percentage
0
25
50
75
c
1C
2C
3C
1H
2H
3H
4H
T-cell composition
100%
75%
50%
25%
0%
1CB 1CT1 1CT2 1CT3
2CB 2CT1 2CT2 2CT3
3CB 3CT1 3CT2
1HB 1HT1 1HT2 1HT3
2HB 2HT1 2HT2 2HT3
3HB 3HT1 3HT2 3HT3
4HB 4HT1 4HT2 4HT3
Tn−CD8
Tn−CD8−activated
Tn−CD8−ISG15
Tn−CD8−LEF1
CD8−IL7R
CD8−S100B
Tpex−CD8
Tem−CD8
Trm−CD8
Tm−CD8−HSPA1A
Tm−CD8−ANXA1
Tm−CD4−HSPA1A
Tm−CD4−ANXA1
Tm−CD4−CD69
Tm−CD4
Treg
CD4−LTB
T−cycling
Tgd
Basophil
Doublet

Supplementary Figure 10. T-cell subsets.

(a) UMAP of T cells colored by different subsets. Tn, native T cell; Tm, memory T cell; Tpex, pre-exhaustion T cell; Tem, effector memory T cell; Trm, tissue resident memory T cell; Treg, regulatory T cell; Tdg, gamma delta T cell. (b) The mean expression and percentage of representative genes in each T-cell subset in (a). The filled color of the circles represents the mean expression of the genes. The size of each circle indicates the proportion of cells in expressing a specific gene. (c) The composition of T-cell subsets in each tumor sample from each case. Source data are provided as a Source Data file.

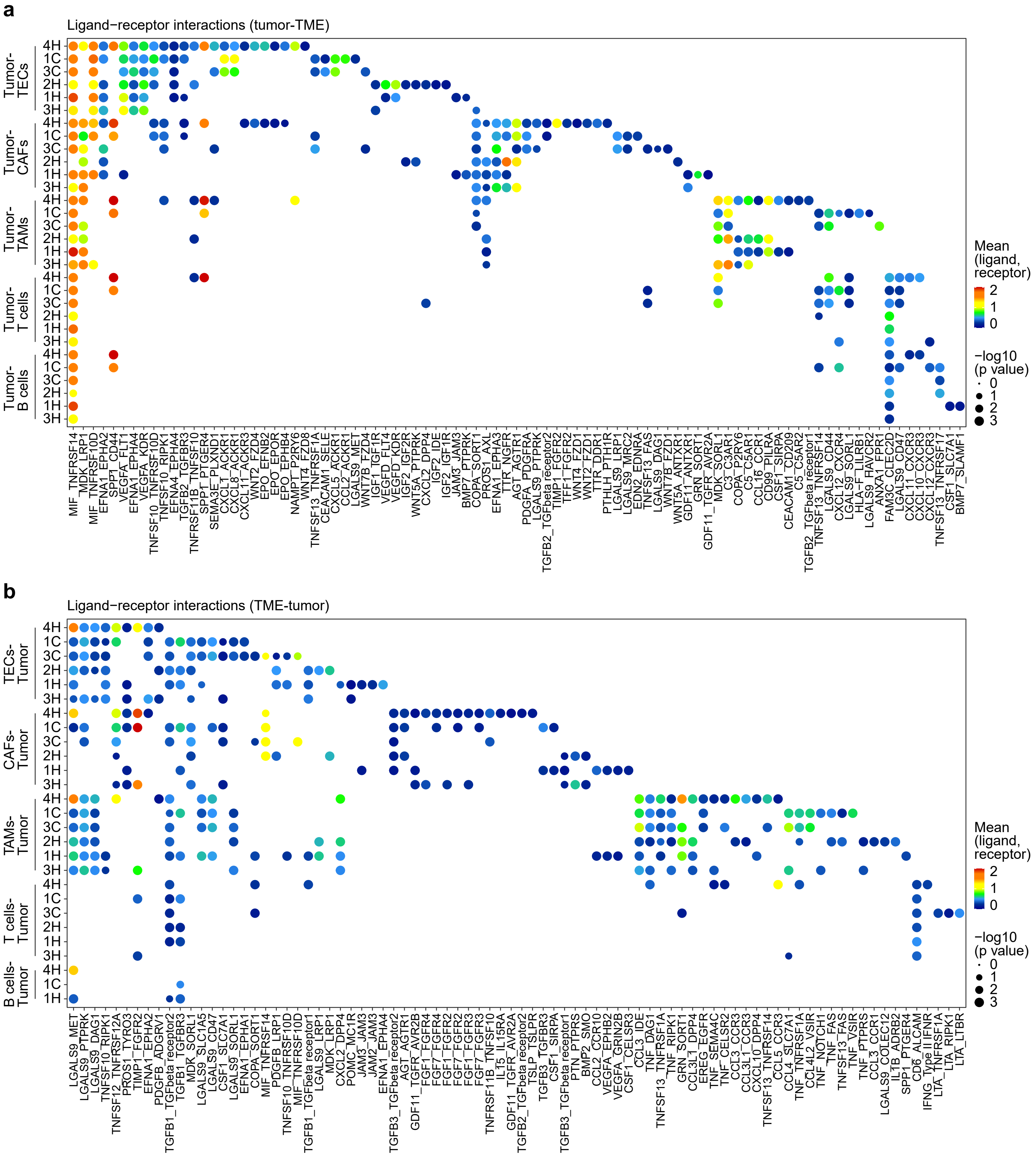
a
Ligand−receptor interactions (tumor-TME)
Tumor-TECs
Tumor-CAFs
Tumor-TAMs
Tumor-T cells
Tumor-B cells
4H
1C
3C
2H
1H
3H
MIF_TNFRSF14
MDK_LRP1
MIF_TNFRSF10D
EFNA1_EPHA2
SPP1_CD44
VEGFA_FLT1
EFNA1_EPHA4
VEGFA_KDR
TNFSF10_TNFRSF10D
TNFSF10_RIPK1
EFNA4_EPHA4
TGFB2_TGFBR3
TNFRSF11B_TNFSF10
SPP1_PTGER4
SEMA3E_PLXND1
CXCL1_ACKR1
CXCL8_ACKR1
CXCL11_ACKR3
WNT2_FZD4
EPO_EFNB2
EPO_EPOR
EPO_EPHB4
NAMPT_P2RY6
WNT4_FZD8
TNFSF13_TNFRSF1A
CEACAM1_SELE
CXCL5_ACKR1
CCL2_ACKR1
LGALS9_MET
WNT7B_FZD4
IGF1_IGF1R
VEGFD_FLT4
VEGFD_KDR
IGF2_IGF2R
WNT5A_PTPRK
CXCL2_DPP4
IGF2_IDE
IGF2_IGF1R
JAM3_JAM3
BMP7_PTPRK
COPA_SORT1
PROS1_AXL
EFNA1_EPHA3
TTR_NGFR
AGT_AGTR1
PDGFA_PDGFRA
LGALS9_PTPRK
TGFB2_TGFbeta receptor2
TIMP1_FGFR2
TFF1_FGFR2
WNT4_FZD1
WNT2_FZD1
TTR_DDR1
PTHLH_PTH1R
LGALS9_LRP1
LGALS9_MRC2
EDN2_EDNRA
TNFSF13_FAS
LGALS9_DAG1
WNT7B_FZD1
WNT5A_ANTXR1
GDF11_ANTXR1
GRN_SORT1
GDF11_TGFR_AVR2A
MDK_SORL1
C3_C3AR1
COPA_P2RY6
C5_C5AR1
CCL16_CCR1
CD99_PILRA
CSF1_SIRPA
CEACAM1_CD209
C5_C5AR2
TGFB2_TGFbeta receptor1
TNFSF13_TNFRSF14
LGALS9_CD44
CXCL12_CXCR4
LGALS9_SORL1
HLA-F_LILRB1
LGALS9_HAVCR2
ANXA1_FPR1
FAM3C_CLEC2D
LGALS9_CD47
CXCL11_CXCR3
CXCL10_CXCR3
CXCL12_CXCR3
TNFSF13_TNFRSF17
CSF1_SLC7A1
BMP7_SLAMF1
Mean (ligand, receptor)
2
1
0
−log10 (p value)
0
1
2
3
b
Ligand−receptor interactions (TME-tumor)
TECs-Tumor
CAFs-Tumor
TAMs-Tumor
T cells-Tumor
B cells-Tumor
4H
1C
3C
2H
1H
3H
LGALS9_MET
LGALS9_PTPRK
LGALS9_DAG1
TNFSF10_RIPK1
TNFSF12_TNFRSF12A
PROS1_TYRO3
TIMP1_FGFR2
EFNA1_EPHA2
PDGFB_ADGRV1
TGFB1_TGFbeta receptor2
TGFB1_TGFBR3
MDK_SORL1
LGALS9_SLC1A5
LGALS9_CD47
CSF1_SLC7A1
LGALS9_SORL1
EFNA1_EPHA1
COPA_SORT1
MIF_TNFRSF14
PDGFB_LRP1
TNFSF10_TNFRSF10D
MIF_TNFRSF10D
TGFB1_TGFbeta receptor1
LGALS9_LRP1
MDK_LRP1
CXCL2_DPP4
POMC_MC1R
JAM3_JAM3
JAM2_JAM3
EFNA1_EPHA4
TGFB3_TGFbeta receptor2
AGT_AGTR1
GDF11_TGFR_AVR2B
FGF1_FGFR4
FGF7_FGFR4
FGF7_FGFR2
FGF1_FGFR2
FGF7_FGFR3
FGF1_FGFR3
TNFRSF11B_TNFSF10
IL15_IL15RA
GDF11_TGFR_AVR2A
TGFB2_TGFbeta receptor2
TSLP_TSLPR
TGFB3_TGFBR3
CSF1_SIRPA
TGFB3_TGFbeta receptor1
PTN_PTPRS
BMP2_SMO
CCL2_CCR10
VEGFA_EPHB2
VEGFA_GRIN2B
CSF1_CELSR3
CCL3_IDE
TNF_DAG1
TNFSF13_TNFRSF1A
TNF_RIPK1
GRN_SORT1
CCL3L1_DPP4
EREG_EGFR
TNF_SEMA4C
TNF_CELSR2
CCL3_CCR3
CCL3L1_CCR3
CXCL10_DPP4
TNFSF13_TNFRSF14
CCL5_CCR3
CCL4_SLC7A1
TNF_TNFRSF1A
CCL4L2_VSIR
TNF_NOTCH1
TNF_FAS
TNFSF13_FAS
TNF_VSIR
TNF_PTPRS
CCL3_CCR1
LGALS9_COLEC12
IL1B_ADRB2
SPP1_PTGER4
CD6_ALCAM
IFNG_Type II IFNR
LTA_TNFRSF1A
LTA_RIPK1
LTA_LTBR
Mean (ligand, receptor)
2
1
0
−log10 (p value)
0
1
2
3

Supplementary Figure 11. Ligand-receptor interactions of malignant cells and non-malignant cells.
(a and b) Ligand-receptor interactions from direction of tumor to TME (a) and TME to tumor (b). Each column indicates a ligand-receptor pair, with the first and the second gene representing a ligand and a receptor, respectively. Each row represents an interaction between two cell types within a case. Color indicates the mean expression value of a ligand-receptor pair. The size of each dot stands for -log10 (p-value) of a specific interaction. p-value was calculated based on 1,000 times of random permutation as described in CellPhoneDB (methods).

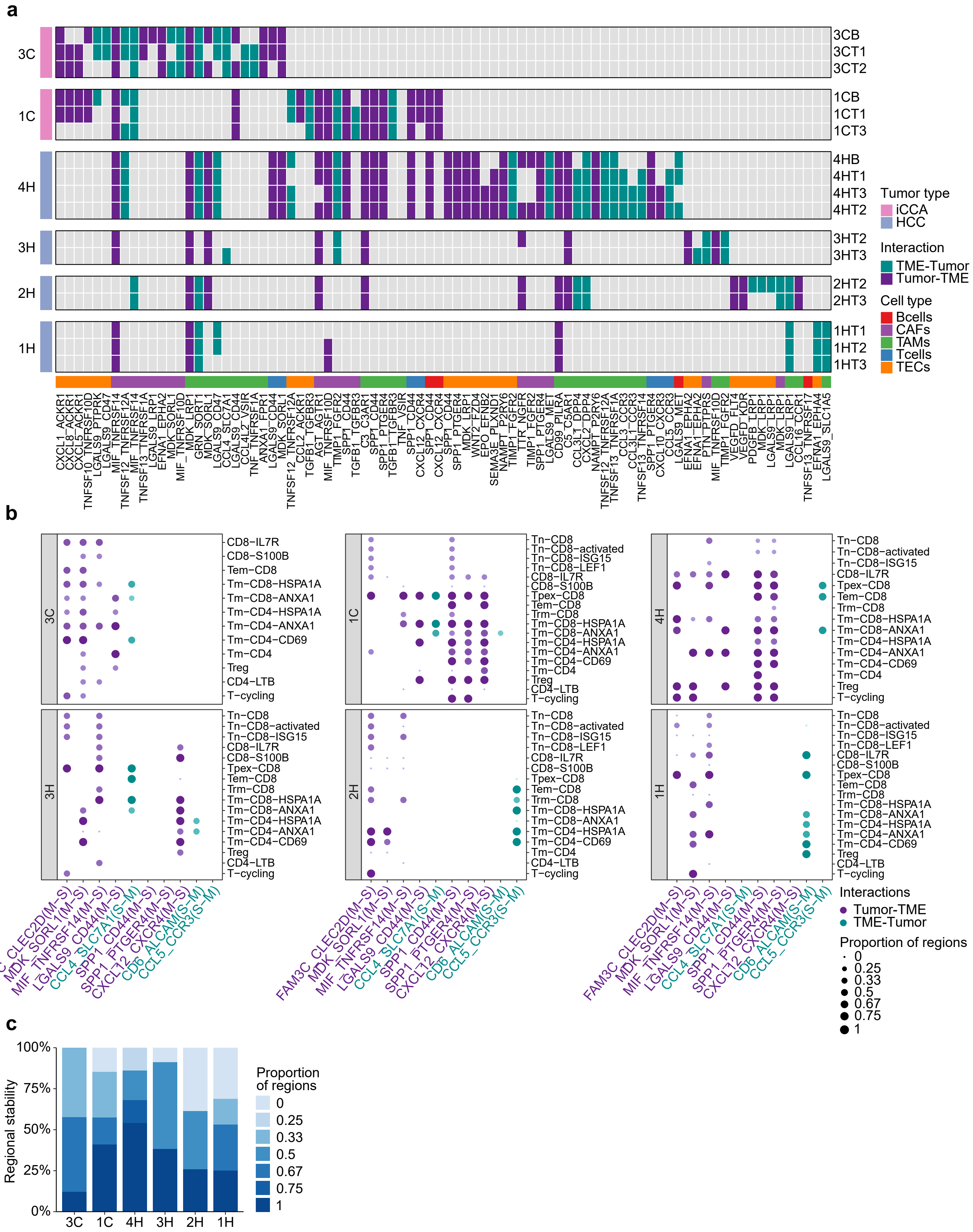
a
3C
1C
4H
3H
2H
1H
3CB
3CT1
3CT2
1CB
1CT1
1CT3
4HB
4HT1
4HT3
4HT2
3HT2
3HT3
2HT2
2HT3
1HT1
1HT2
1HT3
Tumor type
iCCA
HCC
Interaction
TME-Tumor
Tumor-TME
Cell type
Bcells
CAFs
TAMs
Tcells
TECs
CXCL1_ACKR1
CXCL8_ACKR1
CXCL5_ACKR1
TNFSF10_TNFRSF10D
LGALS9_PTPRK
LGALS9_CD47
MIF_TNFRSF14
TNFSF12_TNFRSF12A
MIF_TNFRSF14
TNFSF13_TNFRSF1A
LGALS9_LRP1
EFNA1_EPHA2
MDK_SORL1
MIF_TNFRSF10D
MDK_LRP1
GRN_SORT1
MDK_SORL1
LGALS9_CD47
CCL4_SLC7A1
LGALS9_CD44
CCL4L2_VSIR
TNF_TNFRSF1A
ANXA1_FPR1
LGALS9_CD44
MDK_SORL1
TNFSF12_TNFRSF12A
CCL2_ACKR1
TGFB1_TGFBR3
AGT_AGTR1
MIF_TNFRSF10D
TIMP1_FGFR2
SPP1_CD44
TGFB1_TGFBR3
C3_C3AR1
SPP1_CD44
SPP1_PTGER4
TGFB1_TGFBR3
TNF_VSIR
SPP1_CD44
CXCL12_CXCR4
SPP1_CD44
CXCL12_CXCR4
SPP1_CD44
SPP1_PTGER4
MDK_LRP1
WNT2_FZD4
EPO_EFNB2
SEMA3E_PLXND1
NAMPT_P2RY6
TIMP1_FGFR2
TTR_NGFR
TIMP1_FGFR2
SPP1_PTGER4
LGALS9_MET
CD99_PILRA
C5_C5AR1
CCL3L1_DPP4
CXCL2_DPP4
NAMPT_P2RY6
TNFSF12_TNFRSF12A
TNFSF13_TNFRSF1A
CCL3_CCR3
CCL3L1_CCR3
TNFSF13_TNFRSF14
SPP1_PTGER4
CXCL10_CXCR3
CCL5_CCR3
LGALS9_MET
EFNA1_EPHA2
EFNA1_EPHA2
PTN_PTPRS
MIF_TNFRSF10D
TIMP1_FGFR2
VEGFD_FLT4
VEGFD_KDR
PDGFB_LRP1
MDK_LRP1
LGALS9_LRP1
MDK_LRP1
LGALS9_LRP1
CCL16_CCR1
TNFSF13_TNFRSF17
EFNA1_EPHA4
LGALS9_SLC1A5
b
3C
CD8−IL7R
CD8−S100B
Tem−CD8
Tm−CD8−HSPA1A
Tm−CD8−ANXA1
Tm−CD4−HSPA1A
Tm−CD4−ANXA1
Tm−CD4−CD69
Tm−CD4
Treg
CD4−LTB
T−cycling
1C
Tn−CD8
Tn−CD8−activated
Tn−CD8−ISG15
Tn−CD8−LEF1
CD8−IL7R
CD8−S100B
Tpex−CD8
Tem−CD8
Trm−CD8
Tm−CD8−HSPA1A
Tm−CD8−ANXA1
Tm−CD4−HSPA1A
Tm−CD4−ANXA1
Tm−CD4−CD69
Tm−CD4
Treg
CD4−LTB
T−cycling
4H
Tn−CD8
Tn−CD8−activated
Tn−CD8−ISG15
CD8−IL7R
Tpex−CD8
Tem−CD8
Trm−CD8
Tm−CD8−HSPA1A
Tm−CD8−ANXA1
Tm−CD4−HSPA1A
Tm−CD4−ANXA1
Tm−CD4−CD69
Tm−CD4
Treg
T−cycling
3H
Tn−CD8
Tn−CD8−activated
Tn−CD8−ISG15
CD8−IL7R
CD8−S100B
Tpex−CD8
Tem−CD8
Trm−CD8
Tm−CD8−HSPA1A
Tm−CD8−ANXA1
Tm−CD4−HSPA1A
Tm−CD4−ANXA1
Tm−CD4−CD69
Treg
CD4−LTB
T−cycling
2H
Tn−CD8
Tn−CD8−activated
Tn−CD8−ISG15
Tn−CD8−LEF1
CD8−IL7R
CD8−S100B
Tpex−CD8
Tem−CD8
Trm−CD8
Tm−CD8−HSPA1A
Tm−CD8−ANXA1
Tm−CD4−HSPA1A
Tm−CD4−CD69
Tm−CD4
CD4−LTB
T−cycling
1H
Tn−CD8
Tn−CD8−activated
Tn−CD8−ISG15
Tn−CD8−LEF1
CD8−IL7R
CD8−S100B
Tpex−CD8
Tem−CD8
Trm−CD8
Tm−CD8−HSPA1A
Tm−CD8−ANXA1
Tm−CD4−HSPA1A
Tm−CD4−ANXA1
Tm−CD4−CD69
Treg
CD4−LTB
T−cycling
FAM3C_CLEC2D(M−S)
MDK_SORL1(M−S)
MIF_TNFRSF14(M−S)
LGALS9_CD44(M−S)
CCL4_SLC7A1(S−M)
SPP1_CD44(M−S)
SPP1_PTGER4(M−S)
CXCL12_CXCR4(M−S)
CD6_ALCAM(S−M)
CCL5_CCR3(S−M)
Interactions
Tumor−TME
TME−Tumor
Proportion of regions
0
0.25
0.33
0.5
0.67
0.75
1
c
Regional stability
100%
75%
50%
25%
0%
3C
1C
4H
3H
2H
1H
Proportion
of regions
0
0.25
0.33
0.5
0.67
0.75
1

Supplementary Figure 12. Communication networks of malignant cells and non-malignant cells in multiple tumor regions of each case.

(a) Ligand-receptor interactions of malignant cells and non-malignant cells in six cases with viable malignant cells. Each row represents a tumor region. Each column indicates a ligand-receptor pair, with the first and the second gene representing a ligand and a receptor, respectively. The directions of ligand-receptor interactions are indicated by colors. Purple, malignant cells provide ligands and interact with receptors from non-malignant cells in the TME; green, non-malignant cells in the TME provide ligands and interact with receptors from malignant cells. The non-malignant cell types are indicated by colors. (b) Ligand-receptor interactions between malignant cells and T-cell subsets. Colors represent the directions of the interactions as described in (a). The size of each dot represents the proportion of tumor regions within each case in identifying a specific interaction pair, with 1 indicating occurrence in all tumor regions and 0 indicating occurrence in none of the tumor regions. (c) Stacked bar plot of the percentage of ligand-receptor pairs (between malignant cells and T-cell subsets) in each individual case found in certain proportion of tumor regions. One means that a pair was found in all tumor regions within a case while zero means that a pair was found in none of the tumor regions.

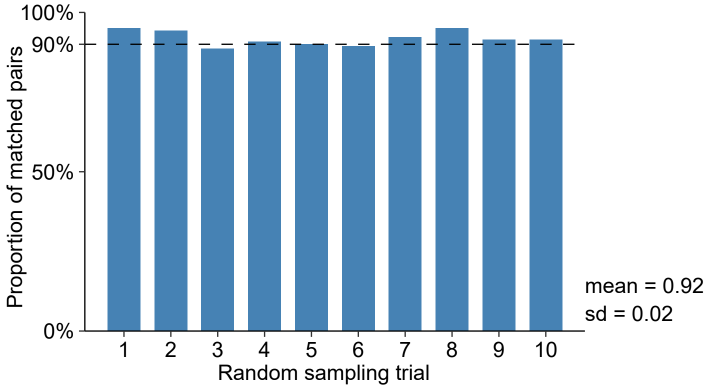

Supplementary Figure 13. Stability test by random sampling T cells and TAMs.
The proportion of matched ligand-receptor interaction pairs by using random selection of T cells and TAMs with the original search using all available single cells. sd, standard deviation.

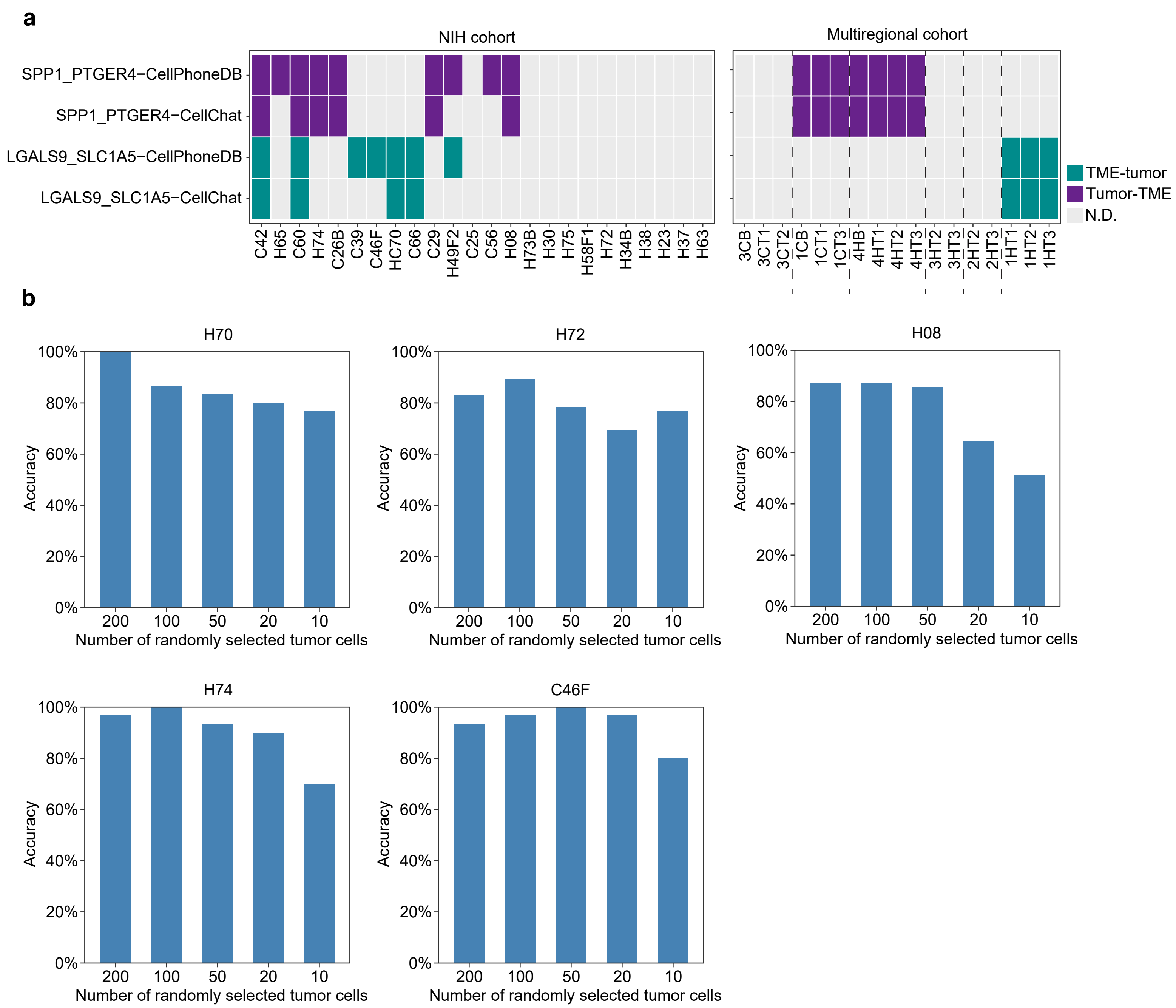

Supplementary Figure 14. Stability test of ligand-receptor pair identification.
(a) Comparison of the ligand-receptor pairs (SPP1-PTGER4, LGALS9-SLC1A5) identified using CellPhoneDB and CellChat in the NIH single-cell cohort and the multiregional single-cell cohort. (b) The accuracy of ligand-receptor pair identification by randomly sampling 200, 100, 50, 20, 10 malignant cells in each case. Accuracy was determined based on five times of random sampling for each setting and further compared with the pairs identified using the original number of malignant cells in Fig. 5a.

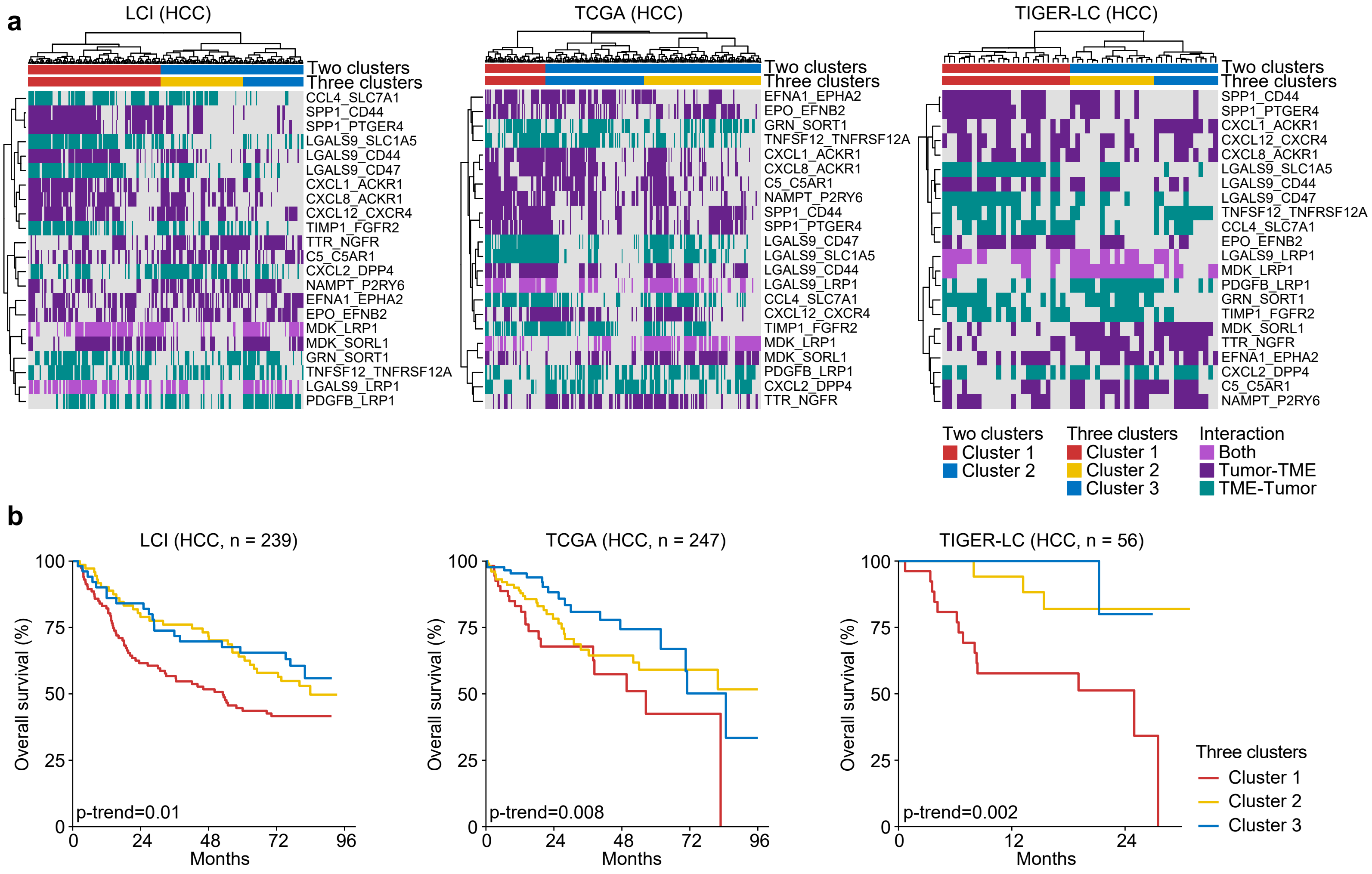

Supplementary Figure 15. Validation of the association between interaction patterns and tumor aggressiveness using bulk transcriptomic data.

(a) Hierarchical clustering of ligand-receptor interaction patterns in three HCC cohorts, i.e., LCI, TCGA, and TIGER-LC. Each row represents a ligand-receptor pair. Each column stands for a tumor sample. The directions of ligand-receptor interactions are indicated by colors. Purple, malignant cells provide ligands and interact with receptors from non-malignant cells in the TME; green, non-malignant cells in the TME provide ligands and interact with receptors from malignant cells; light purple, both directions. Tumor samples were divided into two or three clusters by cutting the hierarchical tree into two or three branches. (b) Overall survival of HCC patients from Cluster 1, Cluster 2 and Cluster 3 determined in (a). The number of samples in each cohort was provided. Log-rank test for trend was preformed to show the statistical significance of the survival trend.

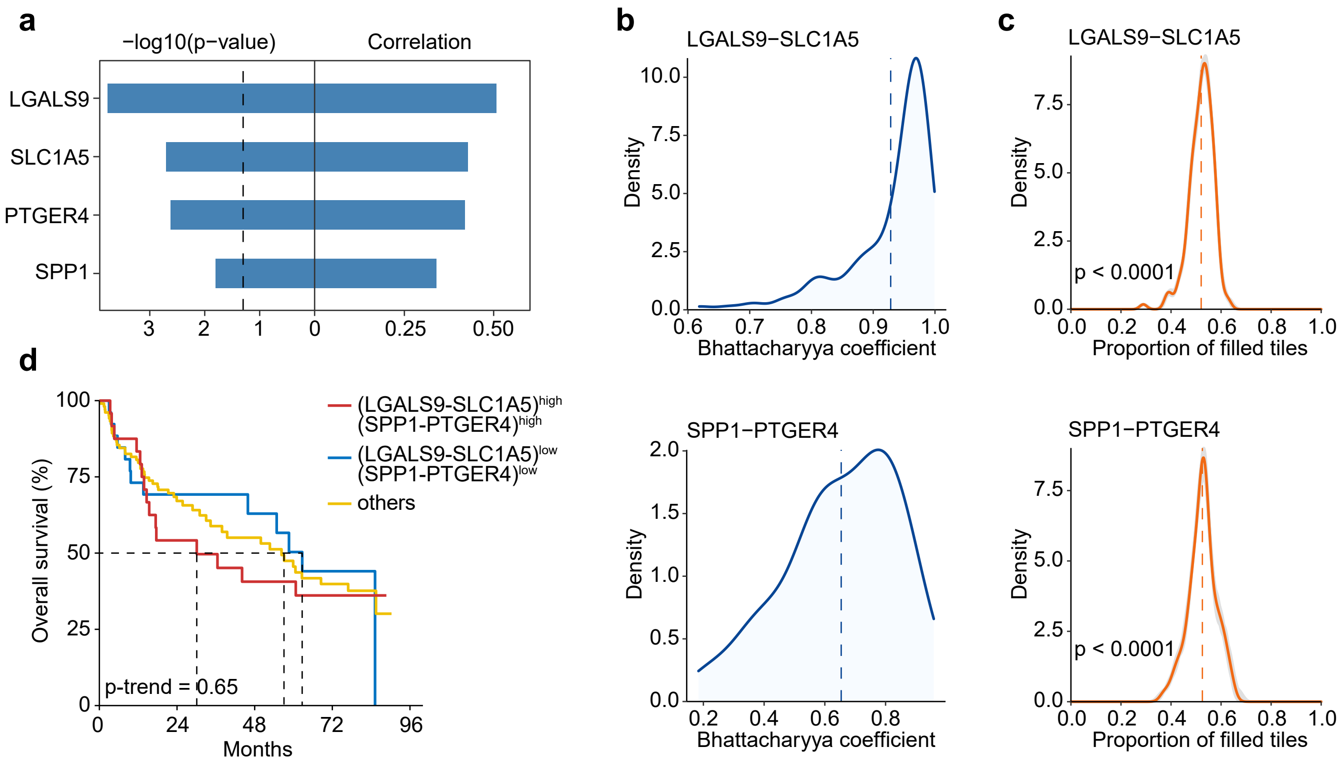

Supplementary Figure 16. RNAscope validation of two interaction pairs in LCI cohort.
(a) Correlation of RNAscope signal and bulk transcriptome gene expression in LCI cohort. Pearson's correlation coefficient (two-sided) was calculated. Dashed line indicates the cutoff of p value in this study, i.e., -log10(p value=0.05). (b) The distribution of Bhattacharyya coefficients of LGALS9 and SLC1A5 (top) as well as SPP1 and PTGER4 (bottom) in HCC samples from LCI cohort. Dashed line indicates the mean value. (c) The distribution of the proportion of filled tiles of LGALS9 and SLC1A5 (top) as well as SPP1 and PTGER4 (bottom) in HCC samples from LCI cohort. Randomization was used to generate the random spread of markers on tissue sections as a reference (see Methods). Gray line, proportion determined based on the ratio of true signal and each random spread; gold line, mean derived from ten gray lines. Dashed line indicates the mean value. p-value is provided to show the statistical difference from 1.(d) Overall survival of HCC patients in LCI cohort with low expression and high expression of LGALS9 and SLC1A5 as well as SPP1 and PTGER4. Tumor samples with the expression of the four marker genes in between were grouped into others. Log-rank test for trend was preformed to show the statistical significance of the survival trend.

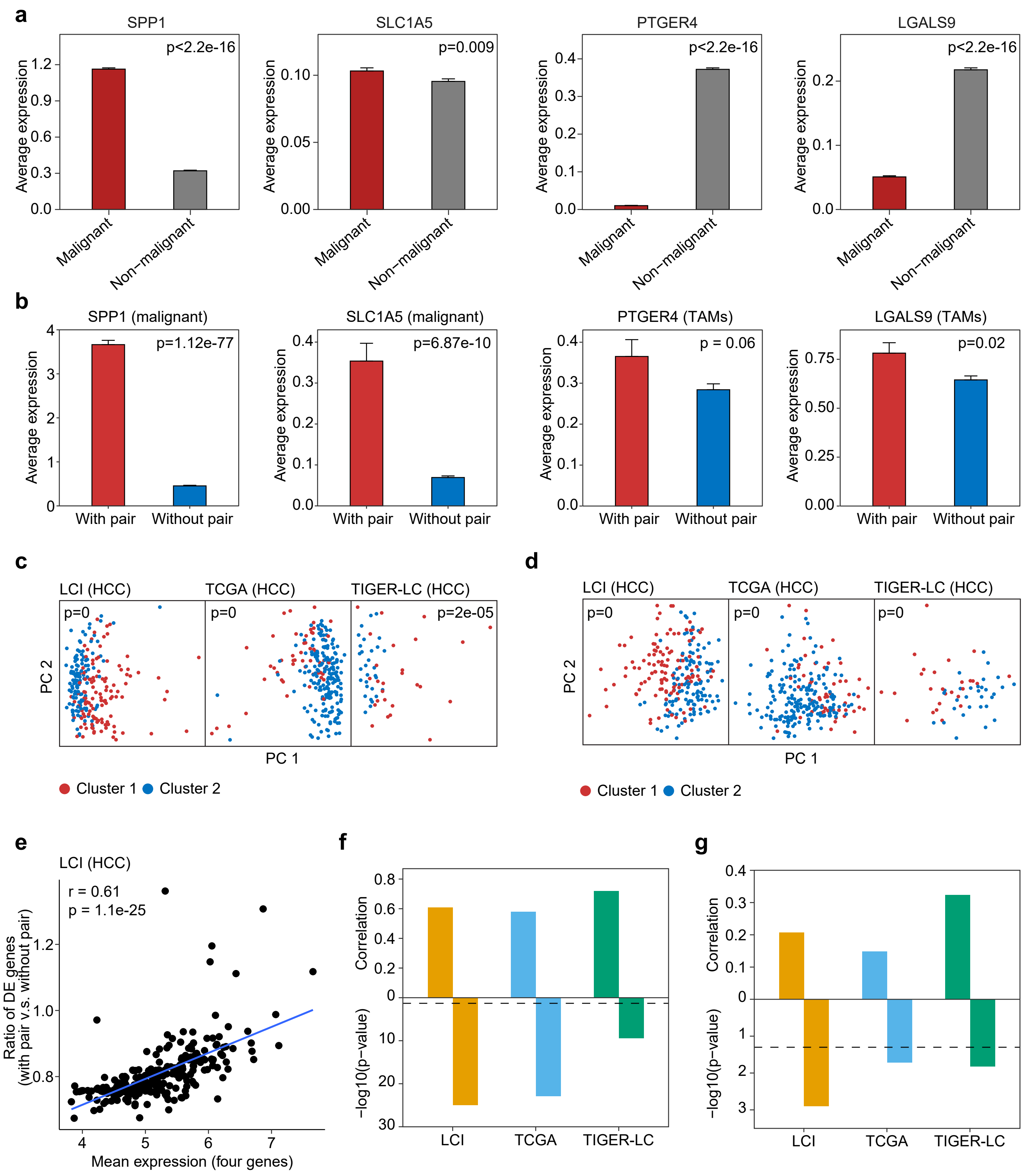
a
SPP1
p<2.2e-16
SLC1A5
p=0.009
PTGER4
p<2.2e-16
LGALS9
p<2.2e-16
Average expression
Malignant
Non−malignant
b
SPP1 (malignant)
p=1.12e-77
SLC1A5 (malignant)
p=6.87e-10
PTGER4 (TAMs)
p = 0.06
LGALS9 (TAMs)
p=0.02
With pair
Without pair
c
LCI (HCC)
TCGA (HCC)
TIGER-LC (HCC)
p=0
p=2e-05
PC 1
PC 2
Cluster 1
Cluster 2
d
e
LCI (HCC)
r = 0.61
p = 1.1e-25
Ratio of DE genes
(with pair v.s. without pair)
Mean expression (four genes)
f
g
Correlation
−log10(p−value)
LCI
TCGA
TIGER-LC

Supplementary Figure 17. Analysis of the two pair (LGALS9-SLC1A5, SPP1-PTGER4) related signaling.
(a) Average expression of SPP1, SLC1A5, PTGER4 and LGALS9 in malignant cells (n=17,164) and non-malignant cells (n=31,154). p value was provided to indicate the statistical significance. Error bar indicates the standard error of the mean. (b) Average expression of SPP1 and SLC1A5 in malignant cells (n=4,235) as well as PTGER4 and LGALS9 in TAMs (n=1,381) in the samples with or without the two pairs (LGALS9-SLC1A5, SPP1-PTGER4) in Fig. 5a. p value was provided to indicate the statistical significance. Error bar indicates the standard error of the mean. (c and d) PCA of tumor samples in three HCC cohorts based on differentially expressed genes of malignant cells (c) or TAMs (d) derived from the samples with or without the two pairs (LGALS9-SLC1A5, SPP1-PTGER4). Cluster 1 and Cluster 2 of the three cohorts were determined in Supplementary Fig.15. Hotelling's T-squared test was used to test the difference of the multivariate means of Cluster 1 and Cluster 2. (e) Correlation analysis in LCI cohort based on the mean expression of the four genes (LGALS9, SLC1A5, SPP1 and PTGER4) and the ratio of average expression of differential expression (DE) genes derived from malignant cells in Fig. 7k. Pearson correlation coefficient (two-sided) and p value are provided. (f) Correlation coefficient and p value in all three HCC cohorts using the same analysis in (e). Dashed line indicates the cutoff of p value in this study, i.e., -log10(p value=0.05). (g) Correlation coefficient and p value in all three HCC cohorts based on similar analysis in (e) but with the DE genes in Fig. 7j. Pearson's correlation coefficient (two-sided) was calculated. Dashed line indicates the cutoff of p value in this study, i.e., -log10(p value=0.05).

Supplementary Table 1. Clinical information of seven primary liver cancer patients involved in this multiregional single-cell study.

Variable	Multiregional single-cell cohort (N=7)
Age[a] — year	
Median	72
Range	63-77
Sex — no. (%)	
Female	3 (42.9)
Male	4 (57.1)
Etiology — no. (%)	
HCV	1 (14.3)
NAFLD	1 (14.3)
NASH	3 (42.9)
None	2 (28.6)
Diagnosis — no. (%)	
HCC	4 (57.1)
iCCA	3 (42.9)
Stage — no. (%)	
I	3 (42.9)
II	3 (42.9)
IVA	1 (14.3)
Vascular invasion — no. (%)	
No	4 (57.1)
Yes	3 (42.9)
Tumor size[b] — cm	
Median	7.5
Range	3-24.5
CA19-9 — no. (%)	
≤35 U/mL	4 (57.1)
>35 U/mL	2 (28.6)
Mssing data	1 (14.3)
Alpha-fetoprotein — no. (%)	
Negative, ≤20 ng/mL	4 (57.1)
Positive, >20 ng/mL	2 (28.6)
Mssing data	1 (14.3)

[a]Age at time of tissue collection used for this study.

[b]Tumor size determined by radiologic measurements.
