# Supplementary material for: Multiregional single-cell dissection of tumor and immune cells reveals stable lock-and-key features in liver cancer
Source: Nat Commun. 2022 Dec 7;13:7533. doi: 10.1038/s41467-022-35291-5 (PMC9729309; doi:10.1038/s41467-022-35291-5)
Supplement: Supplementary file 3 — Description to Additional Supplementary Information [file 41467_2022_35291_MOESM3_ESM.pdf]

Description Of Additional Supplementary Files

Supplementary Data 1: Gene set enrichment analysis of malignant cells or TAMs in tumor samples with or without the two pairs of interactions (LGALS9- SLC1A5, SPP1-PTGER4).
